# Perinatal Exposure to Bisphenol A Alters Early Adipogenesis in the Rat

**DOI:** 10.1289/ehp.11342

**Published:** 2009-06-29

**Authors:** Emmanuel Somm, Valérie M. Schwitzgebel, Audrey Toulotte, Christopher R. Cederroth, Christophe Combescure, Serge Nef, Michel L. Aubert, Petra S. Hüppi

**Affiliations:** 1 Faculty of Medicine, University of Geneva, Geneva, Switzerland; 2 Department of Pediatrics, Geneva University Hospitals, Geneva, Switzerland; 3 Department of Genetic Medicine and Development and National Center for Competence in Research—Frontiers in Genetics, Faculty of Medicine, University of Geneva, Geneva, Switzerland; 4 Department of Clinical Epidemiology, Geneva University Hospitals, Geneva, Switzerland

**Keywords:** adipocyte, adipose tissue, bisphenol A, food intake, obesity

## Abstract

**Background:**

The causes of the current obesity pandemic have not been fully elucidated. Implication of environmental endocrine disruptors such as bisphenol A (BPA) on adipose tissue development has been poorly investigated.

**Objectives:**

The aim of the present study was to evaluate the effects of perinatal exposure to BPA on early adipose storage at weaning.

**Methods:**

Pregnant Sprague-Dawley rats had access to drinking water containing 1 mg/L BPA from day 6 of gestation through the end of lactation. Pups were weaned on postnatal day (PND) 21. At that time, we investigated perigonadal adipose tissue of pups (weight, histology, gene expression). For the remaining animals, we recorded body weight and food intake for animals on either standard chow or a high-fat diet.

**Results:**

Gestational exposure to BPA did not alter the sex ratio or litter size at birth. On PND1, the weight of male and female BPA-exposed pups was increased. On PND21, body weight was increased only in females, in which parametrial white adipose tissue (pWAT) weight was increased about 3-fold. This excess of pWAT was associated with adipocyte hypertrophy and overexpression of lipogenic genes such as *C/EBP-*α (CAAT enhancer binding protein alpha), *PPAR-*γ (peroxisome proliferator-activated receptor gamma), *SREBP-1C* (sterol regulatory element binding protein-1C)*, LPL* (lipoprotein lipase), *FAS* (fatty acid synthase)*,* and *SCD-1* (stearoyl-CoA desaturase 1). In addition, gene expression of *SREBP-1C, FAS*, and *ACC* (acetyl-CoA carboxylase) was also increased in liver from BPA-exposed females at PND21, without a change in circulating lipids and glucose. After weaning, perinatal BPA exposure predisposed to overweight in a sex- and diet-dependent manner. We observed no change in food intake due to perinatal BPA exposure in rats on either standard chow or a high-fat diet.

**Conclusions:**

Perinatal exposure to a low dose of BPA increased adipogenesis in females at weaning. Adult body weight may be programmed during early life, leading to changes dependent on the sex and the nutritional status. Although further studies are required to understand the mechanisms of BPA action in early life, these results are particularly important with regard to the increasing prevalence of childhood obesity and the context-dependent action of endocrine disruptors.

Bisphenol A (BPA), a chemical compound found in plastic products, is increasingly being identified as a pervasive industrial pollutant. In fact, accumulating evidence indicates that the human population is widely exposed to BPA through polycarbonate plastics, resins, and sealants (Brotons et al. 1995; Olea et al. 1996); thus, environmental BPA is an endocrine-disrupting chemical that can potentially affect human health (Kang et al. 2006; vom Saal and Hughes 2005; vom Saal et al. 2005). Importantly, previous studies have detected BPA in serum of pregnant women and in cord serum collected at birth (Ikezuki et al. 2002; Takahashi and Oishi 2000) and reported that BPA accumulates early in fetuses (Schonfelder et al. 2002b). Moreover, BPA content was higher in amniotic fluid and placenta than in maternal serum (Ikezuki et al. 2002; Takahashi and Oishi 2000). *In utero* exposure to BPA has been shown to cause adverse effects in offspring, for example:

- Accelerated puberty and increased body weight in female mice when gestating dams were injected with BPA (2 and 20 μg/kg) (Honma et al. 2002)
- Alterations in rodent mammary gland when dams were implanted with osmotic pumps delivering 25–250 μg/kg/day (Markey et al. 2001)
- Abnormal female genital tract development when dams were exposed to BPA through gavage (Schonfelder et al. 2002a) or osmotic pumps (Markey et al. 2005)
- In males, altered structure and function of the ventral prostate when dams were exposed to BPA through osmotic pump delivering BPA between 25 and 250 μg/kg/day (Ramos et al. 2001).

Mechanistically, BPA is a well-known estrogenic compound that binds to estrogen receptor α (ERα) and ERβ (Hiroi et al. 1999; Kurosawa et al. 2002). BPA can also act as an antagonist of the thyroid hormone receptor (Moriyama et al. 2002; Zoeller et al. 2005) and targets protein disulfide isomerase, a multifunctional protein critically involved in the folding, assembly, and shedding of many cellular proteins (Hiroi et al. 2006; Okada et al. 2005).

The first evidence that perinatal BPA exposure could lead to altered metabolic features was provided by Rubin et al. (2001), who found that perinatal exposure to BPA in Sprague-Dawley rats resulted in an increase in body weight apparent soon after birth and continuing into adulthood. In addition, numerous *in vitro* studies have shown metabolic actions of BPA on adipocyte cell lines. First, BPA interferes with glucose homeostasis. BPA treatment enhanced basal and insulin-stimulated glucose uptake in 3T3-F442A adipocytes because of an increased amount of glucose transporter 4 (GLUT4) protein (Sakurai et al. 2004). Second, BPA triggers adipocyte differentiation: The confluent cultures of 3T3-L1 fibroblasts treated with BPA presented an increase in tri-glyceride content, lipoprotein lipase activity, and glycerol phosphate dehydrogenase activity, suggesting that BPA by itself can promote 3T3-L1 fibroblasts to differentiate into adipocytes (Masuno et al. 2002). 3T3-L1 cells treated with BPA also show increased levels of lipoprotein lipase and adipocyte-specific fatty acid binding protein (aP2) mRNAs, confirming that BPA is able to accelerate the terminal differentiation of 3T3-L1 cells into adipocytes (Masuno et al. 2005). To determine whether perinatal exposure to BPA could have an impact on adipogenesis *in vivo*, thus contributing to the partially unexplained increased prevalence of the metabolic syndrome in industrialized countries, we studied adipose tissue deposition and its profile of gene expression at weaning [postnatal day (PND) 21]. We also monitored food intake and body weight during growth in perinatally BPA-exposed animals fed with a standard chow or a high-fat diet after weaning.

## Materials and Methods

### Animal care, diets, and BPA exposure before weaning

All animals used in this study were treated humanely and with regard for alleviation of suffering. All experimental protocols were approved by the State of Geneva Veterinary Office (Geneva, Switzerland). Virgin female and male genitor Sprague-Dawley rats were purchased from Taconic Europe (Laven, Denmark) and provided with a rodent experimental diet (KLIBA NAFAG 3250; Provimi Kliba, Kaiseraugst, Switzerland) low in phytoestrogens, with genistein content below the detection limit, as stated by the provider. Animals were given this diet 10 days before mating until the end of gestation. We considered the day sperm-positive smears were obtained to be gestation day (GD) 0. Pregnant rats were housed individually under standard conditions (22°C, 12-hr light–dark cycle), with free access to food and water. To mimic the most likely route of human exposure, pregnant females were exposed to BPA at concentrations of 1 mg/L in water beginning on GD6 until the end of lactation (PND21 of offspring), as previously described (Rubin et al. 2001). Control females were given water containing 1% ethanol, the concentration used as a vehicle for the BPA solution. Based on the measurements of the volume reduction in the water bottle, not accounting for possible leakage, evaporation, or spillage, we estimated the mean levels of BPA consumed daily by pregnant females at the end of gestation to be approximately 70 μg BPA/kg/day. Assuming that all water lost from the bottle was consumed, the estimates of the level of BPA exposure may be somewhat higher than actual exposure levels. Water bottles and cages made of polypropylene used for all of these studies were devoid of BPA and analog compounds to avoid potential contamination from sources other than administered drinking water. Tap water locally distributed in our animal facilities is not tested for BPA content, but independent organizations controlling the quality of water in Lake Geneva (from which water originates before physicochemical treatment) evaluated its concentration as approximately 7 ng/L (Edder et al. 2006). We consider the residual BPA content in tap water, after the complex process of filtration, to be too low to interfere with the dose studied. Before PND21, maternal food intake, volume of water consumed, and body weight gain were measured daily. Body weight of offspring was measured on PND1 (at the same time as determination of sex ratio and number of pups per litter) and on PND21.

### Animal care and diets after weaning

On PND21, 2 hr after maternal separation, one cohort of both perinatally BPA-exposed and control animals (*n =* 10 animals per sex in each group, belonging to three different litters of each group) was anesthetized with isoflurane. After animals were weighed and anogenital distance was measured, animals were sacrificed by decapitation. Truncal blood was collected and centrifuged, and plasma was immediately frozen for later analysis. Epididymal white adipose tissue (eWAT) was removed from areas surrounding the epididymis and testis, whereas parametrial white adipose tissue (pWAT) was collected from homologous areas surrounding the uterus. Brown adipose tissue (BAT) was collected from the interscapular region and separated from the attached white adipose tissue. These fat depots, in addition to the liver, were weighed before being frozen in liquid nitrogen and stored at −80°C for later molecular analysis. Adipose depots were selected and dissected using previously described protocols (Ailhaud 2001).

A second cohort of perinatally BPA-exposed and control animals was studied during the postweaning period. On PDN21, these animals were given normal drinking water and were fed *ad libitum* with a standard chow diet (7% of the calories from fat, 76% from carbohydrates, and 17% from proteins) or with a high-fat diet (40% of the calories from fat, 43% from carbohydrates, and 17% from proteins). Metabolizable energy of the standard chow and high-fat diets was 2.6 and 4.3 kcal/g, respectively, as described by the manufacturer (Provimi Kliba). We measured body weight weekly from weeks 4 to 14. Food intake was estimated by the weight of solid pellets placed on grids on top of the poly-propylene cages, which was measured weekly from weeks 8 to 14. We estimated the values to be accurate because we were unable to visually detect pieces of pellet or powder in the bottoms of the cages.

### Histologic examination of adipose tissue

pWAT of 21-day-old BPA-exposed and control rats was fixed in a paraformaldehyde solution for 24 hr and embedded in paraffin. Sections (5 μm) were cut using a microtome (Leica Microsystems, Wetzlar, Germany) and then stained with hematoxylin/eosin. Photographs were taken using an Axiocam camera (Carl Zeiss, Gottingen, Germany).

### RNA preparation and mRNA quantification

Adipose tissue depots and liver were carefully dissected and weighed before being frozen in liquid nitrogen and stored at −80°C. Total RNA was subsequently extracted using Trizol reagent (Invitrogen, Basel, Switzerland), according to the manufacturer’s instructions. We assessed RNA quality using the Agilent RNA 6000 Nanokit with an Agilent 2100 Bioanalyzer (Agilent Technologies, Basel, Switzerland). Samples with inadequate coefficients of purity were excluded from the subsequent analyses (reverse transcription and real-time PCR). We reverse-transcribed 5 μg total RNA using 800 units Moloney murine leukemia virus reverse transcriptase (Invitrogen) in the presence of 0.3 units/μL RNAsin ribonuclease inhibitor (Promega Corp, Madison, WI, USA), 7.5 μM random primers [oligo(dN)6], 1.2 mM dNTP (deoxyribonucleotide triphosphate), and 12 μM DTT (dithiothreitol). The relative expression of transcripts coding for rat *C/EBP*-α (CAAT enhancer binding protein alpha), *PPAR-*γ (peroxisome proliferator-activated receptor gamma), *SREBP-1C* (sterol regulatory element binding protein-1C), *GATA-2* (GATA binding protein 2), *Pref-1* (preadipocyte factor 1), *LPL* (lipoprotein lipase), *ACC* (acetyl-CoA carboxylase), *FAS* (fatty acid synthase), *PPAR-*α (peroxisome proliferator activated receptor-alpha), *PGC-1*α (peroxisome proliferator-activated receptor-γ coactivator 1α), *SCD-1* (stearoyl-CoA desaturase 1), and *GLUT4* were determined by quantitative real-time PCR using an ABI 7000 Sequence Detection System (Applied Biosystems, Applera Europe, Rotkreuz, Switzerland) and were normalized using the ribosomal housekeeping gene *36B4*. We quantified PCR products using the SYBR Green Core Reagent kit (Applied Biosystems). Primers were designed using Primer Express software (Applied Biosystems) and tested for efficiency prior to use. The sequences of the primers used are listed below:

C/EBP-α:

F: AGTTGACCAGTGACAATGACCG;

R: TCAGGCAGCTGGCGGAAGAT

PPAR-γ:

F: CTGACCCAATGGTTGCTGATTAC;

R: GGACGCAGGCTCTACTTTGATC

SREBP-1C:

F: CATCGACTACATCCGCTTCTTACA;

R: GTCTTTCAGTGATTTGCTTTTGTGA

GATA-2:

F: AATCGGCCGCTCATCAAG;

R: TCGTCTGACAATTTGCACAACA

Pref-1:

F: CTGCACTGACCCCATTTGTCT;

R: TTCCCCCGGTTTGTCACA

LPL:

F: ACAGTCTTGGAGCCCATGCT;

R: AGCC

AGTAATTCTATTGACCTTCTTGT

ACC:

F: TCCCGGAGCTACTCTTAAAAAATG;

R: CCCCAACGCCCACATG

FAS:

F: CTCTGGAAGTGCATGCTGTAAGA;

R: GGTAGATGTCATTTGCGAAAGGT

SCD-1:

F: CAACACCATGGCGTTCCA;

R: GCGTGTGTCTCAGAGAACTTGTG

GLUT4:

F: ACTCATTCTCGGACGGTTCCT;

R: CTCCCACATACATAGGCACCAA

PPAR-α:

F: TGGAGTCCACGCATGTGAAG;

R: CGCCAGCTTTAGCCGAATAG

PGC-1α:

F: CTGCCATTGTTAAGACCGAGAA;

R: AGGGACGTCTTTGTGGCTTTT

36B4:

F: TTCCCACTGGCTGAAAAGGT;

R: CGCAGCCGCAAATGC

### Plasma measurements

We measured plasma glucose using the glucose oxidase method (Roche Diagnostics GmbH, Rotkreuz, Switzerland). Plasma nonesterified fatty acid and triglyceride levels were determined using kits from Wako Chemicals GmbH (Neuss, Germany) and Biomérieux (Marcy l’Etoile, France), respectively. Plasma cholesterol levels were measured as described by James and Pometta (1990).

### Glucose tolerance test

For the glucose tolerance tests, 14-week-old male rats, perinatally exposed to BPA, and controls, both fed with a high-fat diet since weaning, were fasted from the previous day and injected intraperitoneally with 1.5 mg glucose per gram of body weight. Blood samples were collected by tail puncture before and at selected time points (0, 15, 30, 60, and 120 min) after glucose administration in order to measure glycemia using a glucose meter (Glucotrend Premium, Roche Diagnostics).

### Statistics

Results are expressed as mean ± SEM. We performed the unpaired Student’s *t*-test and repeated-measures one-way analysis of variance (ANOVA) using SYSTAT 10.01 (SPSS Inc., Chicago, IL, USA). We considered a *p*-value of < 0.05 statistically significant. The statistical analyses were performed using litter as the fundamental unit of comparison when appropriate and animals originated from at least three different litters in each group (eight litters for each group for data at birth). To specifically assess the significance of the difference in birth weight and weaning weight between the control and BPA groups, we also used a regression model, taking the correlation between the pups from the same litter into account [generalized estimating equation (GEE) with an exchangeable structure for the working correlation matrix]. The factors introduced in the model were group, sex, litter size, and the interaction between the sex and group.

## Results

### Maternal physiology during gestation

To evaluate whether BPA exposure during gestation affected maternal physiology, we measured weight gain and food and water intake of exposed dams during pregnancy. The weight gain throughout the pregnancy was not significantly different in the BPA-exposed dams (135 ± 7 g; mean ± SEM) compared with control dams (140 ± 6 g; *p* = 0.56). Total food intake was similar in BPA-exposed dams (431 ± 15 g) and control dams (428 ± 16 g; *p =* 0.87) between the start of BPA exposure (GD6) and parturition. The daily volume of water consumed was unaltered by absence or presence of BPA in the water, whatever the stage of gestation considered. In fact, at GD7, control dams drank 24 ± 1 mL and BPA dams drank 25 ± 1 mL (*p =* 0.76). At GD14, both control and BPA dams drank 26 ± 1 mL (*p =* 0.83), and at GD21, control dams drank 29 ± 2 mL and BPA dams drank 30 ± 2 mL (*p =* 0.88). Based on these measurements, we estimated the mean levels of BPA consumed daily by pregnant females from the drinking water to be approximately 30 μg BPA/dam at the end of gestation. Assuming that each gestating dam weighed approximately 428 ± 11 g before parturition, we estimated the total BPA exposure to be approximately 70 μg/kg/day at the end of gestation. Bottle leakage, evaporation, or spillage could result in actual BPA exposure being lower than our calculated levels, which represent a maximal estimation.

### Body weight of offspring at birth, sex ratio, and litter size

**Figure 1:**
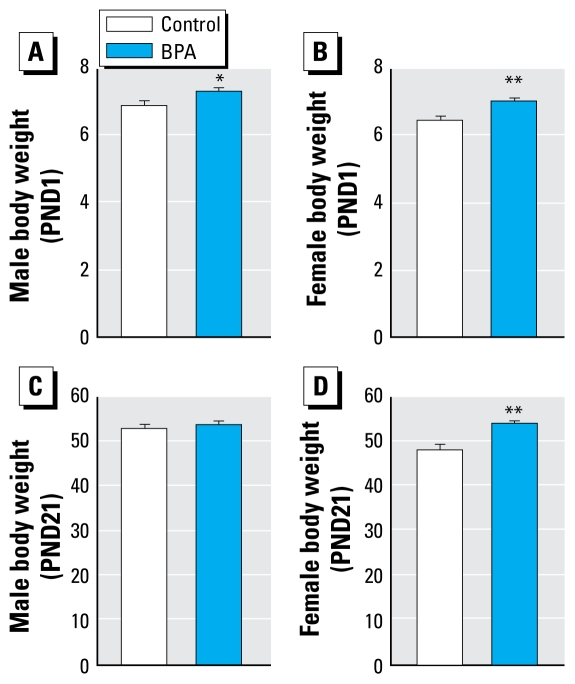
Effect of perinatal exposure to 1 mg/L BPA on body weight of rat pups at PND1 (*A*, males; *B*, females) and PND21 (*C*, males; *D*, females). Results are mean ± SEM. In (*A*), *n =* 45 BPA-exposed males from eight litters; *n =* 55 control males from eight litters. In (*B*), *n* = 50 BPA-exposed females from eight litters; *n* = 47 control females from eight litters. In (*C*), *n =* 32 BPA-exposed males from six litters; *n =* 40 control males from six litters; *p =* 0.59. In (*D*), *n* = 32 BPA-exposed females from six litters; *n =* 26 control females from six litters. *p < 0.05, and **p < 0.001, compared with controls.

On day 1, body weight of male pups born to BPA-exposed dams was significantly higher (7.33 ± 0.12 g, *n =* 45 from eight litters) compared with those of male pups born to control dams (6.91 ± 0.15 g, *n =* 55 from eight litters; *p* < 0.05). The body weight of female pups born to BPA-exposed dams was higher on day 1 (7.03 ± 0.11 g, *n =* 50 from eight litters) than that of pups born to control dams (6.47 ± 0.12 g, *n =* 47 from eight litters; *p* < 0.001) (Figure 1A,B). Despite the fact that the number of pups per litter was not statistically different between the groups (BPA, 12.7 ± 1.0, *n =* 8 litters; control, 14.1 ± 0.9, *n =* 8 litters; *p =* 0.16), the elevation of body weight in pups prenatally exposed to BPA could have been an indirect effect of BPA via a slight reduction of litter size. However, some arguments refute this hypothesis. First, when we compared only size-matched litters, for analysis (*n* = 13.5 and 14 animals per litter in the control and BPA groups, respectively), body weight of day-1 male and female pups born to BPA-exposed dams remained higher than that of controls (BPA, 7.01 ± 0.08 g, *n =* 54 animals from four litters; control, 6.44 ± 0.08 g, *n =* 56 animals from four litters; *p <* 0.001). Moreover, when we used a regression model, adjusted on the litter size, to analyze these data (GEE), the day 1 body weight of BPA-exposed animals remained higher, both for male (*p* < 0.001) and female (*p* < 0.001). Taken together, these observations argue in favor of a direct effect of BPA on body weight of newborn rats, independent of litter size. Finally, we observed that the sex ratio of newborn pups was not altered by prenatal BPA exposure: The proportion of males per litter was similar in control (53 ± 4%) and in BPA-exposed animals (50 ± 4%; *p =* 0.31).

### Body weight and fat pad weights at weaning

Because offspring were exposed to BPA through lactation, we repeated the body weight measurements of these animals at weaning. On PND21, the body weight of BPA-exposed male rats (53.36 ± 1.02 g, *n =* 32) was not different from that of controls (52.54 ± 1.11 g, *n =* 40; *p =* 0.59) (Figure 1C). In contrast, the body weight of BPA-exposed female rats remained higher (53.73 ± 0.65 g, *n =* 32) than that of controls (47.79 ± 1.44 g, *n =* 26; *p <* 0.001) (Figure 1D). When the GEE regression model was used to analyze these data, the interaction between sex and group was significant (*p* = 0.02), and for females, the BPA-induced elevation of body weight was statistically significant after adjustment for litter size (*p* = 0.001).

**Figure 2:**
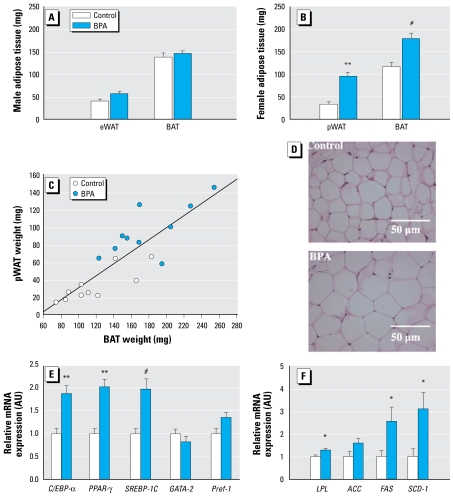
Effect of perinatal exposure to BPA on adipogenesis at weaning (PND21). (*A*) Weight of eWAT and BAT in male rats. (*B*) Weight of pWAT and interscapular BAT in female rats; data represent 10 animals from three independent litters. (*C*) Correlation between pWAT and BAT weight in female rats (*n =* 10 animals per group; *r*^2^ = 0.74; *p <* 0.001). (*D*) Representative histologic sections of pWAT from control (top) and BPA-exposed females (bottom). (*E*, *F*) Relative mRNA levels [in arbitrary units (AU)] in pWAT of female rats at weaning (*n =* 9–10 animals in each group from three litters). Results shown in *A, B*, *E*, and *F* are mean ± SEM. *p < 0.05, **p < 0.001, and ^#^*p* < 0.002, compared with controls.

To study the potential impact of BPA exposure on body composition, we sacrificed a fraction of these animals at weaning (PND21) and weighed two different fat pads: inter-scapular BAT and perigonadal white adipose tissue (WAT), also called eWAT in males and pWAT in females. Interestingly, we found an increased trend of eWAT mass in male pups exposed to BPA (56 ± 6 mg) compared with controls (39 ± 6 mg; *p =* 0.06), whereas this reached significance in BPA-exposed female pWAT (95 ± 9 mg) compared with controls (33 ± 6 mg; *p <* 0.001) (Figure 2A,B). BAT weight was unchanged in males (Figure 2A) but was significantly higher in females exposed to BPA (178 ± 13 mg) compared with controls (116 ± 11 mg; *p =* 0.002) (Figure 2B). Moreover, in females, BAT weight correlated with pWAT weight (*r*^2^ = 0.74, *p <* 0.001) (Figure 2C). These observations suggest that the increase in body weight in BPA-exposed females is, in part, related to an increase in adipose depot weight. In contrast, no change was observed in weight (relative to body weight) of organs such as heart, liver, or kidney (data not shown). Morphologically, we observed no difference in male anogenital distance (AGD) (14.8 ± 0.5 mm for controls vs. 16.1 ± 0.7 mm for BPA animals; *p =* 0.18), but we saw a slight increase in female AGD (8.45 ± 0.4 mm for controls vs. 10 ± 0.3 mm for BPA animals; *p <* 0.01) (data not shown). Overall, these results suggest that females tend to be more sensitive than males to the BPA-induced fat gain at the dose tested.

### Expression of genes involved in adipogenesis and lipogenesis in adipose tissue at weaning

To better characterize the early increase in white adipose tissue deposition observed in perinatally BPA-exposed females pups, we performed histologic sections of their pWAT. As shown in representative photographs of these tissues (Figure 2D), adipocytes of rats perinatally exposed to BPA appeared to be hypertrophied compared with those of control animals. These histologic observations correlated with changes in the expression of genes involved in metabolism. mRNA levels of the proadipogenic transcription factors C/EBP-α, PPAR-γ, and SREBP-1C were significantly increased [by 1.87 ± 0.18-fold (*p* < 0.001), 2.02 ± 0.17-fold (*p* < 0.001), and 1.97 ± 0.23-fold (*p* < 0.002), respectively] in pWAT of BPA-exposed females at weaning (Figure 2E). In contrast, mRNA levels of inhibitors of adipogenesis, such as GATA-2 and Pref-1 remained unchanged (Figure 2E). Gene expression levels of lipogenic enzymes such as LPL, FAS, and SCD-1 were up-regulated [by 1.28 ± 0.09-fold (*p* < 0.05), 2.55 ± 0.63-fold (*p* < 0.05), and 3.10 ± 0.73-fold (*p* < 0.05), respectively] in pWAT of BPA-exposed females (Figure 2F). mRNA levels of GLUT4 were also increased by 1.59 ± 0.25-fold (*p* < 0.05; data not shown).

Taken together, these findings strongly suggest that perinatal exposure to BPA enhances adipogenesis and lipogenesis *in vivo* through an increase of the transcription of genes implicated in adipocyte metabolism.

### Expression of genes involved in lipogenesis in liver at weaning

**Figure 3:**
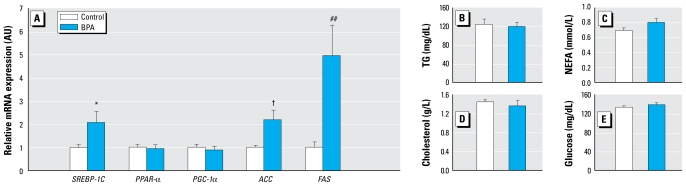
Effect of perinatal exposure to BPA on gene expression in liver and circulating metabolites in females at weaning (PND21). (*A*) Relative mRNA levels [in arbitrary units (AU)] in liver (*n =* 10 animals per group from three litters). Results are means ± SEM. (*B–E*) Circulating levels of TG (*B*), NEFA (*C*), total cholesterol (*D*), and glucose (*E*) in plasma (*n =* 10 animals per group from three litters). *p < 0.05, ^##^*p <* 0.01, and ^†^*p <* 0.02, compared with controls.

Liver is a key organ involved in metabolism, as it controls synthesis of many nutrients including lipids and carbohydrates. We measured hepatic change in expression of several metabolic genes. Messenger RNA levels of SREBP-1C were significantly increased by 2.07 ± 0.47-fold (*p <* 0.05) in the liver of BPA-exposed females at weaning (Figure 3A). In contrast, no change was observed in gene expression of the transcription factors PPAR-α and PPAR-gamma-coactivator 1 alpha (PGC1-α), involved in fatty acid oxidation and gluconeogenesis, respectively, in this organ. mRNA levels of prolipogenic enzymes such as ACC and FAS were also up-regulated by 2.17 ± 0.43-fold (*p <* 0.02) and 4.94 ± 1.33-fold (*p <* 0.01), respectively, in the liver of BPA-exposed females (Figure 3A). To assess whether these early alterations in gene expression were related to changes in circulating levels of nutrients, we measured the levels of triglycerides (TG), nonesterified fatty acid (NEFA), glucose, and total cholesterol in females pups at weaning. We found no statistical difference in circulating TG (control, 123.2 ± 12.1 mg/dL; BPA 118.8 ± 9.6 mg/dL; *p =* 0.84; Figure 3B), NEFA (control, 0.69 ± 0.04 mmol/L; BPA 0.80 ± 0.06 mmol/L; *p =* 0.18; Figure 3C), cholesterol (control, 1.45 ± 0.05 g/L; BPA 1.36 ± 0.02 g/L; *p =* 0.13; Figure 3D), and glucose levels (control, 135.1 ± 3.3 mg/dL; BPA 140.6 ± 4.5 mg/dL; *p =* 0.29; Figure 3E).

### Postweaning body weight

**Figure 4:**
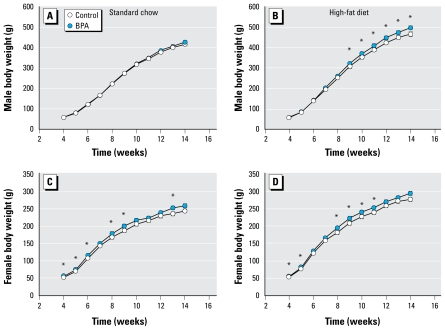
Effect of a high-fat diet on body weight of male (*A, B*) and female (*C, D*) rats perinatally exposed to BPA. . Animals were fed standard chow (*A, C*) or the high-fat diet (*B, D*) during weeks 4–14; *n =* 8–12 animals per group. Note the smallest curve of body weight in *A* and *C* (chow diet) compared with *B* and *D* (high-fat diet). *p < 0.05 compared with control,

After weaning, we weighed perinatally BPA-exposed animals weekly from 4 weeks to 14 weeks of age. We found no statistical difference in body weight between perinatally BPA-exposed males and control males on the standard chow diet (Figure 4A). However, perinatally BPA-exposed males on a high-fat diet were heavier than controls, and a difference of 7 ± 2% of body weight at 14 weeks of age (*p <* 0.05) was observed (Figure 4B), suggesting that a long-term effect of BPA exists in males only when combined with a high caloric intake. In contrast, in females we observed differences in body weight between perinatally BPA-exposed animals and control animals on both standard chow and high-fat diets (Figure 4C,D). Overall, these results suggest that perinatal exposure to BPA increases the susceptibility to weight gain in a sex-specific manner, even after weaning.

### Postweaning food intake and glucose tolerance at adulthood

To evaluate whether differences in body weight observed after perinatal BPA exposure are related to altered appetite regulation, we measured food intake of perinatally BPA-exposed and control rats on the standard chow or the high-fat diet from week 8 to week 14. During the 7 consecutive weeks of measurement, despite the known caloric hyperphagia induced by the high-fat diet, we detected no impact of perinatal BPA exposure on daily food intake or cumulative energy intake in males or females eating either diet. Perinatally BPA-exposed males on the standard diet ingested 3,331 ± 116 kcal (vs. 3,308 ± 54 kcal for controls; *p =* 0.85), whereas perinatally BPA-exposed males on the high-fat diet consumed 3,931 ± 52 kcal (vs. 3,854 ± 111 kcal for controls; *p =* 0.57). Likewise, perinatally BPA-exposed females on the standard diet ingested 2,358 ± 58 kcal (vs. 2,251 ± 7 kcal for controls; *p =* 0.15), whereas perinatally BPA-exposed females on the high-fat diet consumed 2,655 ± 81 kcal (vs. 2,510 ± 31 kcal for controls; *p =* 0.20). Therefore, perinatal exposure to BPA did not affect postweaning food intake during growth, either in standard conditions or on a high caloric diet.

To determine whether the difference in body weight in BPA-exposed males fed a high-fat diet had an impact on glucose homeostasis, we performed glucose tolerance tests in both BPA-exposed and control males on a high-fat diet. Basal glucose levels after 16 hr of fasting were similar in control rats (4.63 ± 0.10 mmol/L) and BPA-exposed rats (4.65 ± 0.08 mmol/L; *p =* 0.88). After intraperitoneal administration of glucose, the circulating glucose clearance was similar in both groups (data not shown), reflecting no disturbance in glucose utilization. This was confirmed by measuring the area under the curve, which was equivalent in both BPA-exposed (1,312 ± 89 mmol/L × min) and control animals (1,319 ± 97 mmol/L × min; *p =* 0.96). These results suggest that perinatal exposure to BPA has no long-term effects on glucose metabolism in the male rat on a high-fat diet.

## Discussion

Many studies have investigated the long-term impact of early BPA exposure during stages of tissue organization on the male reproductive tract (Prins et al. 2006; Watanabe et al. 2003), the female reproductive tract (Suzuki et al. 2002; Vandenberg et al. 2007), and behavior (Aloisi et al. 2002; Della Seta et al. 2005; Dessi-Fulgheri et al. 2002; Farabollini et al. 2002; Fujimoto et al. 2006; Porrini et al. 2005). However, until recently, little data on the metabolic effects of perinatal BPA exposure were available. The doses we tested (1 mg/L in drinking water, corresponding to a maximal 70 μg BPA/kg/day exposure at the end of gestation, based on water consumed), as well as the oral route of exposure chosen (similar to exposure through plastic and resin in contact with food), render this model relevant for humans. Despite the fact that this dose cannot be considered truly environmentally relevant, it can be considered low (Chapin et al. 2008; Melnick et al. 2002).

Maternal physiology (assessed by body weight gain, food intake, and volume of water consumed) of the BPA-exposed gestating dams was unchanged, as were the sex ratio of pups born and number of pups per litter, as previously reported (Honma et al. 2002; Rubin et al. 2001).

In the present study, we confirmed that the body weight at birth of both male and female pups prenatally exposed to BPA was significantly increased. At weaning, after postnatal BPA exposure via milk during lactation, the body weight of BPA-exposed females, but not males, remained higher than controls. These observations are in accordance with some previous publications demonstrating increased postnatal growth in different rodent species exposed to maternal BPA doses between 2.4 and 500 μg/kg/day (Howdeshell et al. 1999; Nikaido et al. 2004; Rubin et al. 2001; Takai et al. 2001). However, our present data are in contradiction with others works reporting no alteration of body weight in pups exposed to a very wide range of maternal BPA doses, between 0.001 and 5 mg/kg/day (Tyl et al. 2002Tyl et al. 2008). Conversely, in these toxicity studies, higher doses of BPA administered to gestating dams (50–600 mg/kg/day) reduced body weight in growing Sprague-Dawley rat and CD-1 mouse pups (Tyl et al. 2002Tyl et al. 2008).

In our study we observed that the increase in offspring body weight due to BPA exposure through placenta and milk was associated with an increase in the early adipose storage at weaning in a sex-dependent manner. After perinatal exposure to BPA, eWAT demonstrated a trend to increase in males, and pWAT was increased by nearly 3-fold in females. Our observations in Sprague-Dawley rats are in agreement with a recent report in which gonadal fat pads were heavier in ICR female mice exposed continuously until 30 days of age to the same dose of BPA that we used (Miyawaki et al. 2007). In the same study, Miyawaki et al. (2007) noted that a 10-fold higher dose was inefficient in inducing overstorage of fat in females but was efficient in increasing fat storage in males. This led the authors to conclude that BPA caused a nonmonotonic and inverted U-shaped dose–response increase in adipose tissue weight in females. Sex-dependent susceptibility to xenoestrogens are highly specific for a given window of exposure and the kind of xenoestrogens used. For example, direct oral exposure to genistein (an isoflavone with estrogenic activities found mostly in soy) in 4-week-old mice increased weights of male but not female fat pads (Penza et al. 2006), whereas *in utero* exposure to genistein protected A(vy/a) mice from obesity in association with methylation changes of the epigenome (Dolinoy et al. 2006). In the same A(vy/a) mouse model, *in utero* exposure to BPA at 50 mg/kg has also demonstrated epigenetic effects, as it decreased CpG methylation in an intracisternal A particle retrotransposon upstream of the Agouti gene (Dolinoy et al. 2007).

In the present study, we also illustrate for the first time that pWAT enlargement at weaning in females exposed perinatally to BPA is associated with adipocyte hypertrophy, an expanding tissue process more easily reversible than hyperplasia, which corresponds to an increase in preadipocyte recruitment. We show that change in size of adipocytes in BPA-exposed females is associated with an overexpression of proadipogenic transcription factors (C/EBP-α, PPAR-γ, SREBP-1C). These *in vivo* observations are in accordance with previous *in vitro* observations, as BPA by itself can accelerate the differentiation of 3T3-L1 fibroblasts into adipocytes (Masuno et al. 2002). The gene expression of lipogenic enzymes (LPL, FAS, SCD-1) was also up-regulated in the WAT of BPA-exposed females, corroborating the *in vitro*–increased levels of lipoprotein lipase mRNAs of 3T3-L1 cell culture treated with BPA (Masuno et al. 2005). Finally, we observed an increase in mRNA levels of *GLUT4* in WAT of BPA-exposed females, in agreement with the *in vitro*–enhanced glucose uptake due to an increased amount of GLUT4 protein, which was observed in 3T3-F442A adipocytes treated with BPA (Sakurai et al. 2004). The molecular pathways implicated in these pro-adipogenic effects of BPA are unknown, but it has been suggested that the PI3K/Akt pathway could be involved (Masuno et al. 2005), as BPA increased the level of phosphorylated Akt kinase, and LY294002, a chemical inhibitor of PI3K, completely abolished the enhancing effect of BPA on triglyceride accumulation and expression of prolipogenic mRNAs. Finally, we show that perinatal BPA exposure is associated with an overexpression of lipogenic transcription factor and enzymes (SREBP-1C, ACC, FAS) in the liver. Again, these *in vivo* observations are in accordance with previous *in vitro* observations in which BPA increased lipogenesis through glycerol accumulation in the HuH-7 hepatocellular cell line (Wada et al. 2007). At PND21, we found no difference in circulating levels of triglyceride, nonesterified fatty acid, and glucose. We also failed to detect changes in cholesterol levels in perinatally BPA-exposed pups, contrary to those observed in ICR mice (Miyawaki et al. 2007). However, in that study, measurements were done at PND30, after consumption of a high-fat diet and direct exposure of pups to BPA via drinking bottle.

Taken together, these findings provide the first clear direct evidence of early gene expression alterations in adipose tissue and liver after perinatal BPA exposure—an interesting observation, as prevalence of childhood obesity is increasing worldwide (Laron 2004; Micic 2001; Reilly 2005), and some countries, such as Canada, prohibit the use of BPA in babies’ bottles.

In our study, animals were only indirectly exposed through placenta and milk, and BPA exposure was stopped at weaning. To uncover a possible programming effect of BPA persisting beyond the end of exposure, we investigated possible metabolic disturbances after weaning. First, we observed heavier body weight during growth in females perinatally exposed to BPA, independent of the diet provided (standard chow or high-fat diet), in line with results previously reported by Rubin and al. (2001). More surprisingly, BPA males presented increased body weight during growth only when placed on a high-fat diet, suggesting that BPA predisposition to overweight is potent only when food is hypercaloric.

A possible explanation of enhanced weight gain in BPA-exposed animals is an increase in food intake. In fact, estrogenic action can affect neuronal circuits that control appetite by acting on the hypothalamus (Nunez et al. 1980; Wade and Schneider 1992). Moreover, based on its detection in the brain, BPA is known to cross the brain–blood barrier (Sun et al. 2002). In this way, we investigated postweaning food intake in perinatally exposed animals fed standard chow or a high-fat diet. No changes in food intake were observed in male or female animals perinatally exposed to BPA compared with their respective controls. As body weight and energy storage result from a balance between energy intake and energy expenditure, this latter parameter could more probably explain the difference in body weight observed in response to BPA. It should be noted that in addition to its estrogenic activities, BPA binds and antagonizes the thyroid hormone receptor and therefore inhibits transcriptional activity stimulated by triiodothyronine (Moriyama et al. 2002; Zoeller 2005; Zoeller et al. 2005). It is conceivable that perinatal BPA exposure disrupts the thyroid hormone axis, which itself plays a predominant role in thermogenesis and energy expenditure. It is interesting to note the preferential accumulation of BPA in BAT (Nunez et al. 2001), a major mediator of thyroid hormone on thermogenesis. Additional studies are required to better understand the precise role of BPA in energy expenditure and thermogenesis. Growth hormone could also be involved in increased body weight gain in relation to perinatal BPA exposure, as it has recently been shown that BPA induced growth hormone release *in vitro* in the GH3 pituitary cell line (Okada et al. 2007).

In conclusion, the present study demonstrates that direct exposure to BPA through placenta and milk increases early adipose storage and adipogenesis in a sex-specific manner, confirming previous observations in ICR mice (Miyawaki et al. 2007) and *in vitro* in 3T3-L1 and 3T3-F442A adipocyte cell lines (Masuno et al. 2002, 2005; Sakurai et al. 2004). Increased adipose storage caused by perinatal BPA exposure could be a major public health concern in relation to the epidemic of childhood obesity (Laron 2004; Micic 2001; Reilly 2005). Moreover, because exposure to BPA is ubiquitous and does not cease during human life (Brotons et al. 1995), further studies are urgently needed to better understand the long-term consequences of permanent BPA exposure on body weight homeostasis.
